# The role of otorhinolaryngologists in the treatment of hereditary hemorrhagic telangiectasia

**DOI:** 10.1186/s13005-025-00567-w

**Published:** 2025-12-13

**Authors:** René Lehner, Robin Lochbaum, Thomas K. Hoffmann, Jens Greve

**Affiliations:** Department of Otorhinolaryngology, Head and Neck Surgery, University Hospital of Ulm, Frauensteige 12, Ulm, 89075 Germany

**Keywords:** HHT, Epistaxis, Arteriovenous malformations, ORL

## Abstract

**Purpose:**

Hereditary hemorrhagic telangiectasia (HHT) is an autosomal dominant disease which affects the entire vascular system. Symptoms vary from mild complaints to severe complications and are highly individual. Therapy is strictly symptomatic and creates an interdisciplinary challenge with relevant involvement of otorhinolaryngologists (ORL). The aim of this study is to evaluate this involvement.

**Methods:**

A retrospective analysis between 01/2006 and 12/2023 at Ulm University Medical Center was conducted. Two collectives were defined, the first one representing all patients who have been treated in any department for HHT, in order to draw a comparison between the departments. The second focused specifically on patients treated in the ORL department, providing a detailed analysis that includes emergency visits, hospital admissions, interventions and blood transfusions.

**Results:**

In the 1^st^ collective 252 patients were identified generating 1092 cases. 43.3% of the patients were primarily treated at the ORL department, 24.0% at the dermatology department and 13.1% in internal medicine. The ORL patients generated significantly more cases as other specialties (*p* = 0.0001). The 2nd collective consisted of 105 patients. Men were more likely to be hospitalized (*p* = 0.0001) and to receive blood transfusions (*p* = 0.0045). There were no other gender specific differences. Symptoms and the need for medical help were independent of the season. A vast variety of visits, admissions and interventions could be shown.

**Conclusion:**

ORL specialists play a critical role in the management of HHT patients. The study shows the year-round necessity for specialized care. Sex-related differences should be taken into account. Besides multidisciplinary therapy strategies further prospective research is essential.

## Introduction

Hereditary hemorrhagic telangiectasia (HHT), also known as *Osler’s disease* (or *Rendu-Osler-Weber-Syndrome*), is an autosomal dominant disease which affects the vascular system. The prevalence ranges between 1:5000 and 1:10000 worldwide, though local discrepancies can occur [1, 2]. Due to the genetic disorder, the blood vessels in the entire body can change and lead to mostly small, cutaneous or mucosal telangiectasias or even bigger arteriovenous malformations (AVM) in the brain, lung, and liver [3].

**Fig. 1:**
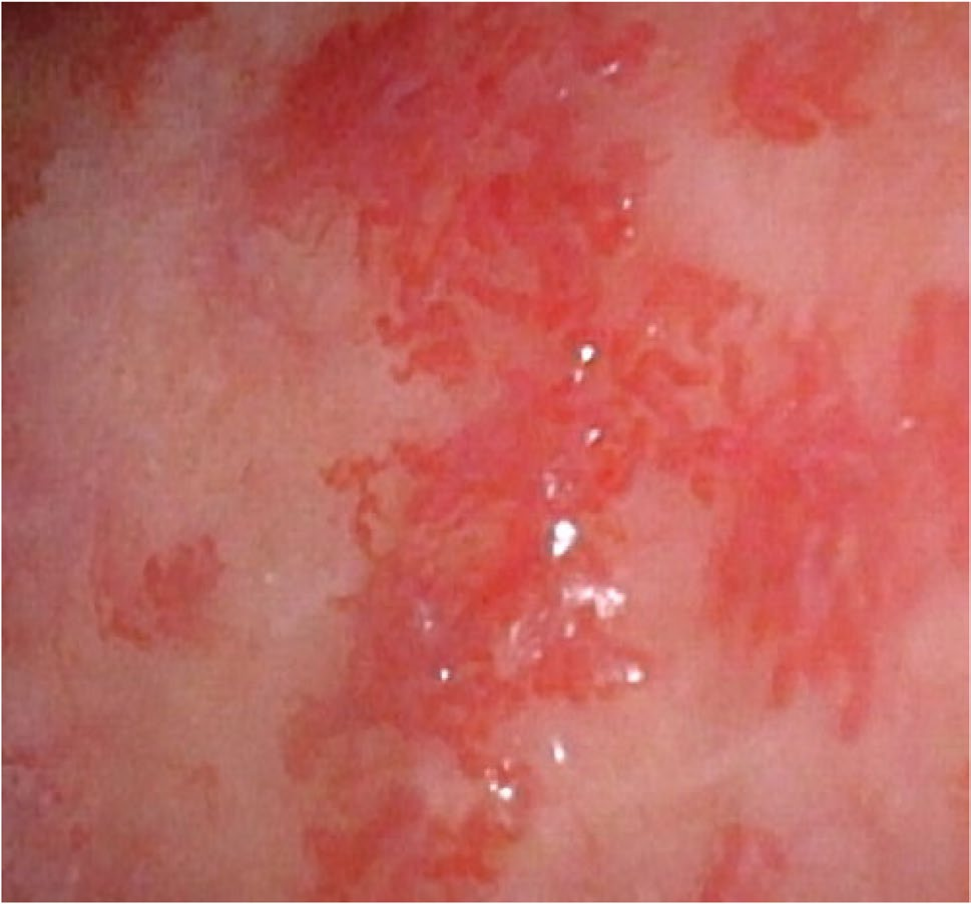
Endonasal mucosa with typical spots

Intensity of complaints varies from cosmetically disruptive telangiectasias to recurrent epistaxis to paradoxical embolisms and abscesses and is different in each patient [3]. However, almost every patient presents the main symptom: epistaxis. More than 90% suffer from recurrent nosebleeds, often unaware of the cause. These two facts (epistaxis and a family history of such) and typical spots (Fig. 1) which mostly occur in the face, enoral and endonasal as well as at the fingertips can help to find the diagnosis by using the *Curaçao-Criteria* [4]. Genetic testing is not necessarily required but can help determine the HHT type depending on the pathologic sequence variant in the specific genes.

There is still no causal therapy. All therapeutic approaches are symptomatic. Concerning the main symptom, epistaxis, therapy is based on local measures, which are primarily intended to guarantee moistening of the nasal mucosa in order to achieve a good mucosal condition and to prevent further bleeding. The four basic measures are (1) the regular use of ointments, (2) water-based nasal sprays and inhalation, (3) nasal irrigation, and (4) temporary nasal occlusion [5]. Often, the basic treatments do not suffice to maintain a tolerable level of epistaxis. To evaluate the local control and to objectify the symptoms, the *Epistaxis* or *Nosebleed Severity Score (ESS/NSS)* can be calculated [6]. Local laser therapy is the therapy of choice [7]. Due to the recurring nature of the spots, multiple interventions are required. Moreover, a recurrence of the complaints must be expected.

Additional manifestations, beside the head and neck, are common, especially pulmonary and hepatic involvements [8]. A screening regime for patients and first-degree relatives for AVMs is recommended because of possible severe complications [9].

Since the disease can affect every vessel and, by that can have a variety of clinical appearances, as well as the fact that serious complications can occur, the disease is often misdiagnosed [10]. Usually, the therapy is an interdisciplinary challenge [11]. Therefore, most patients need a well-established team of physicians.

The aim of this study was to provide a comprehensive overview of the specific characteristics of patients with HHT. The survey sought to explore the potential scope of necessary treatment upon diagnosis of HHT. It was hypothesized that the disease causes year-round symptoms, with no seasonal variation, unlike conditions such as common nosebleeds, which tend to fluctuate seasonally [12]. As epistaxis is the primary symptom of HHT, it was further hypothesized that these patients would be more likely to seek care in the ORL department compared to other specialties. Gender and age-related differences should be investigated as well.

## Materials & methods

This study was conducted as a retrospective observational cohort study.

The investigation and analysis were carried out at the ORL department of Ulm University Medical Center between 01/2006 and 12/2023, thus spanning 18 years. All relevant data was obtained in this timeframe. The inclusion criteria were simply defined by the ICD-10 (International Classification of Diseases) code for HHT: I78.0. Patients without this coding were not included. The *Curaçao-Criteria* and/or genetic testing determined the diagnosis.

Two separate collectives have been defined:

The first consisted of all HHT patients who had been treated at any department of the Ulm University Medical Center. The completely anonymous data collection was carried out by the general controlling. The data obtained in this way consisted solely of the number of patients and the case numbers of the respective departments. A case refers to any visit by a department and patient within a 3-month period (“quarter”) and does not reflect the exact number of actual visits. Further personal and medical data was not collected for data protection reasons. Coding errors cannot be ruled out, as the data collection was conducted by a third party. Coding errors may include false diagnosis and the creation of multiple patient IDs, e.g., in emergency situations, thus leading to different total numbers.

The second collective equals the number of HHT patients of the ORL department. This analysis was conducted using the department’s own electronic medical records, which have been the standard of documentation since 2006. With this tool all relevant data from the ORL department can be accessed. It contains anamneses, clinical examinations, surgery and transfusion reports. Therefore, the exact number of visits as well as the number of surgical interventions could be analyzed for ORL patients. Thereby, any intervention from laser therapy to permanent nasal occlusion was considered. In addition, a clear distinction was made between regular visits and emergency visits. An emergency visit was defined as one in which the patient needed immediate medical attention that could not be managed independently. This included situations such as acute bleeding that the patient was unable to control, as well as cases of severe exhaustion and fatigue that required urgent care. However, bleedings that occurred earlier, e.g., the day before the visit, were not considered emergencies. Moreover, the month in which the visits occurred and the need for blood transfusions were also taken into account. This allowed for a more accurate assessment of the types of visits and to identify a seasonal variation.

Data was collected and statistically analysed using Microsoft Excel (version 1808), Prism 5/GraphPad. Descriptive and nonparametric statistical analyses were performed. For group comparison, Fisher’s exact test was mainly used, for mean analysis the unpaired t-test was applied. To compare observed and expected frequencies the chi-square test was used. A p-value of 0.05 or less was considered statistically significant.

Ethics approval of the local ethical committee was obtained beforehand without the need of written consent due to the retrospective approach (application number: 225/20).

## Results

### 1st collective

**Fig. 2:**
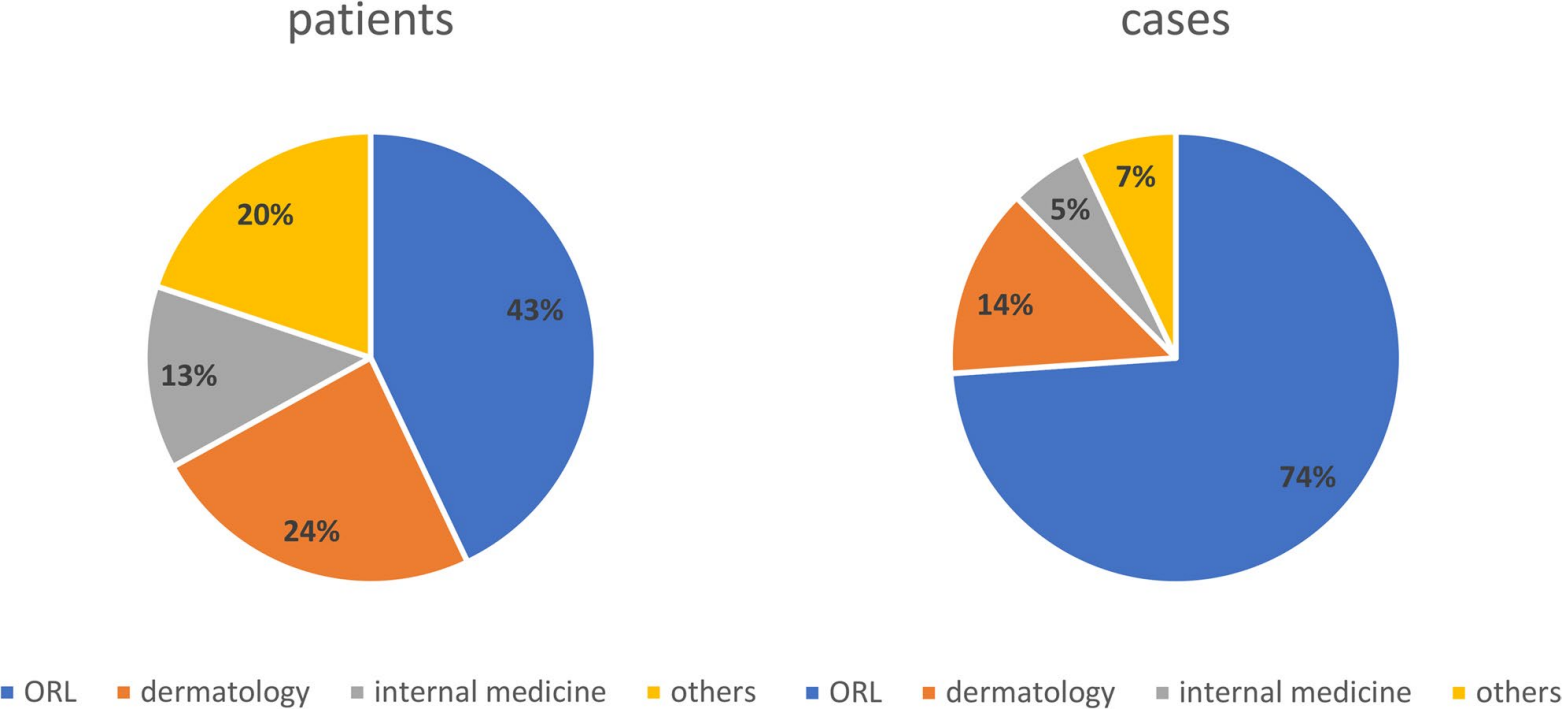
Relative numbers of cases and patients in the different departments

In the first collective, which consisted of all HHT patients treated at Ulm Medical Center, 252 patients were identified. They generated 1092 cases in 13 different departments, an average of 4.3 cases per patient. 215/252 [85.7%] patients were treated in one department, 20/252 [7.9%] in two, 13/252 [5.2%] in three, 3/252 [1.2%] in four and 1/252 [0.4%] in six different departments. Only 35/252 [13.9%] patients had multiple visits to other departments than the ORL. For mathematical reasons a total of 312 department visitations (corresponds to the sum of patients being multiplied by the number of departments visited) was used for the following analysis:

Most patients were seen in the ORL department (134/312[43.3%]), followed by dermatology (75/312 [24.0%]) and internal medicine (IM) (41/312 [13.1%]). ORL patients generated 808/1092 [74.0%] cases, dermatology patients 149/1092 [13.6%] and IM patients 59/1092 [5.4%]. When comparing the expected number of cases based on patient volume to the actual observed cases, there was a significantly higher number of cases in the ORL department than in others (*p* = 0.0001, see Fig. 2).

### 2nd collective

#### Clinical and demographic features

The second collective consisted of 105 patients (m: 42/105 [40%]; f: 63/105 [60%]). This collective equals a detailed analysis of the patients from the ORL department. The average age at first contact was 51.3 years, the average therapy duration 4.81 years. 95/105 [90.5%] HHT diagnoses were based on the *Curaçao-Criteria* alone. Genetic test results were available for the remaining 10 patients. 5/105 [4.8%] were classified as HHT-Type 1, 4/105 [3.8%] as HHT-Type 2 and one patient carried the SMAD4-mutation [1.0%]. 217 times the ESS/NSS were calculated, the average score was 4.74.

In total 1,278 visits had been documented. Based on the therapy duration, this results in an average of 2.62 visits per year and patient; the median is 2.18, the first quartile is 1.02 and the third quartile is 3,1. 259/1,278 [20.3%] visits occurred as an emergency and were generated by 37/105 [35.2%] patients. 181/1,278 [14.2%] visits resulted in a hospitalization, 145 of those admissions being due to emergency situations. 33/105 [31.4%] patients were admitted at least once. 17/105 [16.2%] patients required blood transfusions, leading to a total of 75 transfusions. 66/105 [62.9%] patients had one or more interventions, in total 476 interventions were accounted for. 395/476 [83.0%] were laser-therapies, the other 81/476 [17.0%] included septal splints, septodermoplasty, endonasal arterial clipping, and the *Young’s* procedure (total nasal closure.)

#### Gender and age comparison

Men were responsible for 609/1,278 [47.7%] and women for 669/1,278 [52.3%] visits. When comparing the means there was a slight tendency towards women generating more visits, though with no statistical significance (m = 50.75, f = 55.75; *p* = 0.0562). When considering emergency visits, there was no difference between men and women (m = 11.08, f = 10.50; *p* = 0.6757).

703/1,278 [55.0%] visits were generated by patients under the age of 60, 757/1,278 [45.0%] by older patients (*p* = 0.5786). There was no statistical difference between men and women (*p* = 0.5741). Furthermore, there was no statistically significant difference in emergency visits, admissions and blood transfusions when comparing patients over and under 60 years of age.

Men had a total of 119 admissions, averaging 9.92 admissions per patient. In contrast, women were admitted 62 times, with an average of 5.17 admissions per patient, which was statistically significantly lower than that of men (*p* = 0.0001). There were 145 emergency admissions in total, with men averaging at 7.50 and women at 4.58 (*p* = 0.0045). There was the necessity for blood transfusions 75 times, men averaging at 4 transfusion per patient, women at 2.25 transfusions per patient (*p* = 0.0073).

Men generated 185/476 [38.9%] interventions, averaging at 4.4 interventions per patient. Women on the other hand received 291/476 [61.1%] interventions with an average of 4.62. There was no statistically significant difference (*p* = 0.8583). An overview of the results is given in Table 1.

**Table 1:** Overview gender comparison with absolute numbers and *p*-values, *statistical significant values are bolded

	Men	Women	*p*-value
Total visits	609	669	0.0562
Visits under the age of 60	340	363	0.5741
Visits over the age of 60	269	306	
Emergency visits	133	126	0.6757
Total admissions	119	62	0.1000*
Emergency admissions	90	55	0.0045*
Blood transfusions	48	27	0.0073*
Interventions	185	291	0.8583

#### Seasonal dependence

**Fig. 3:**
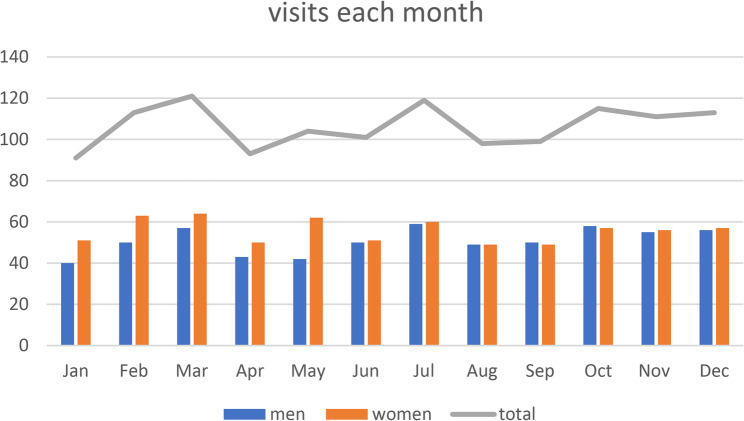
Total number of visits each month for men, women and both

A total of 1278 visits were documented and are graphically displayed in Fig. 3 for seasonal dependence.

The observed number of visits did not differ significantly from the expected ones (*p* = 0.4756). This was also the case for the gender comparison (*p* = 0.6119 for men; *p* = 0.8539 for women). Emergency visits were also equally distributed with no difference in gender either.

**Fig. 4:**
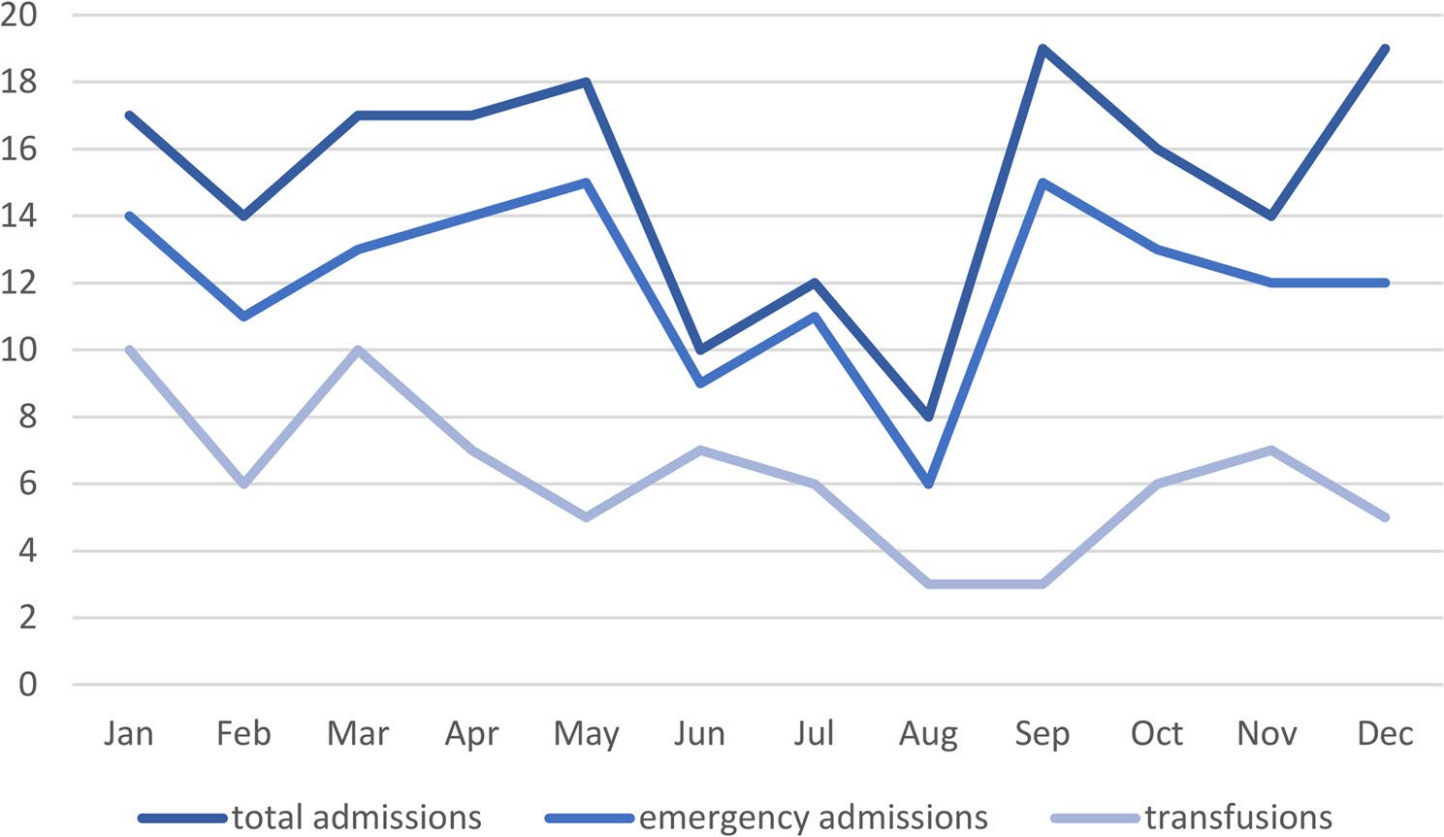
Total number of admissions, emergency admissions and blood transfusions during each month

Furthermore, there was a normal distribution in admissions (*p* = 0.6025), emergency admissions (*p* = 0.8597) and blood transfusions (*p* = 0.6514). From June to August there are fewer admissions, but without statistical significance (Fig. 4). No gender differences were observed.

**Fig. 5:**
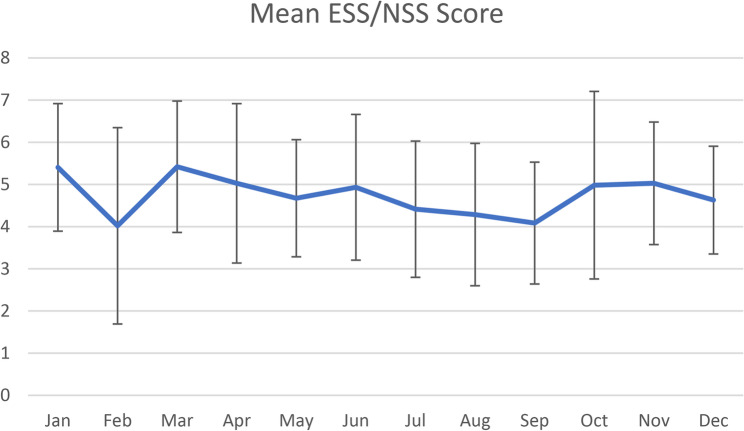
Mean ESS/NSS scores each month based on the total of 217 recorded scores, with standard deviations

641/1,278 [50.2%] visits occurred from November until April, 637/1,278 [49.5%] from May until October. There was no significant difference in those groups (*p* = 0.7508), neither when comparing genders. The analysis of the ESS/NSS scores in the same timeframe (Nov-Apr and May-Oct) did not show a statistical difference either (*p* = 0.7276), neither did the gender analysis (*p* = 0.4834) (Fig. 5).

#### Frequencies

36/105 [34.4%] patients had 1 to 2 visits, 69/105 [65.7%] had more than 2 visits. 68/105 [64.8%] patients had no need for an emergency visit, 37/105 [35.2%] on the other hand did so. 72/105 [68.6%] have never been hospitalized, 33/105 [31.4] at least once or more often. 88/105 [83.8%] did not need blood transfusions, 17/105 [16.2%] patients received at least one or more transfusions. 39/105 [37.1%] had no interventions, 66/105 [62.9%] did. In Table 2 further information is shown. There were no statistically significant differences between men and women.

**Table 2:** Overview of the number of visits, emergency visits, admissions, blood transfusions and interventions as a percentage of the total number of patients (*n* = 105) as well as the lowest and highest number in each category

*n*	total visits	emergency visits	admissions	transfusions	interventions
0		64.8%	68.6%	83.8%	37.1%
1 to 2	34.3%	14.3%	12.4%	5.7%	26.7%
3 to 5	20.0%	4.8%	9.5%	6.7%	16.2%
6 to 10	18.1%	6.7%	4.8%	1.0%	8.6%
11 to 20	14.3%	6.7%	3.8%	2.9%	6.7%
21 to 50	8.6%	2.9%	1.0%		2.9%
51 to 100	1.0%				1.9%
> 100	3.8%				
Minimum	1	0	0	0	0
Maximum	115	23	24	13	58

## Discussion

The involvement of otorhinolaryngologists in the management of HHT is described in one of the most thorough ways to date by this large single-center cohort. With a total of 252 patients and a lengthy follow-up period of 18-years, our findings contribute significant nuance to the vast variety of different clinical appearances of HHT patients. While confirming well known facts, such as the predominance of epistaxis, sex related differences were able to be identified.

### ORL is the core specialty in HHT management

Our findings show that, in both acute and long-term settings, ORL departments continue to lead the way in HHT care. Although this result is in line with earlier recommendations and analyses [3, 7, 13], our longer observation period emphasizes the cumulative and ongoing burden of epistaxis over almost 20 years. The high number of hospitalizations and interventions highlights the necessity of well-organized interdisciplinary centers under ORL lead, which are rarely discussed in the literature currently in publication.

### Sex related differences

Male patients had a much higher rate of hospitalization and transfusion, which was an unexpected and noteworthy finding. Our data indicates that male sex may be a risk factor for more severe disease manifestations, as few previous studies have examined sex-specific differences in HHT severity. To our knowledge, there is none describing similar differences. Hormonal influences, biological variations in vascular fragility, or variations in healthcare-seeking behavior are some possible explanations. Even though our retrospective design cannot draw firm conclusions, this observation merits verification in prospective research and may eventually help guide risk assessment and customized follow-up procedures.

### Seasonality

No seasonal variation in visits related to (HHT-)epistaxis was observed. As it shows the ongoing disease activity throughout the year, this result has clinical significance. Seasonal variation in common epistaxis may be a well-known fact, that has been addressed in other studies before [14, 15], but the lack of seasonal effects in HHT patients has not. It does have practical ramifications for patient counseling and hospital resource allocation.

### Health system implications

The combined cost of hospital stays, transfusions, and interventions highlights the significant healthcare resources needed for HHT care. Our results support the development of interdisciplinary care centers, as mentioned before. Furthermore, centralized documentation and structured follow-up may minimize care variation and improve results.

### Future studies

Due to the rarity of the disease and the expertise required for its treatment, multicenter studies are essential in order to achieve relevant changes in understanding and therapy. Epistaxis scores, documentation of sex-specific differences and genetic analysis should be part of future data collection and may be achieved through a national database. This may help future research set the focus on targeted therapy strategies, as they are already available for other genetic diseases [16–18]. With this achievement sophisticated evidence-based algorithms may be created for optimal and individual treatment.

### Limitations

There are various limitations in this study. Potential biases are due to its retrospective design, which includes documentation heterogeneity and incomplete data. Although we made an effort to minimize double counting, some analyses may have included inflated numbers due to the combination and creation of hospital-wide (1st collective) and ORL specific (2nd collective) cohorts. Additionally, the majority of our analysis was descriptive. The documentation of the ESS/NSS was not common in the early years of the data acquisition and only just became part of the general data collection with increasing patient numbers and expertise. The score calculation, or more the anamnesis leading to said score, can underlie individual differences. For that, the analysis of the ESS/NSS must be looked at with caution. The mentioned limitations must be noted when reading this single-center study. Some of those may have been able to reduce in a multicenter approach.

## Conclusion

This research highlights the critical role that ORL specialists play in managing HHT. It suggests that the disease burden may differ by sex, and shows that ORL care is required all year round, regardless of the season. In addition to supporting the creation of organized, multidisciplinary management strategies and highlighting the chronic healthcare demands posed by HHT, these findings also indicate the necessity of prospective, outcome-oriented research.

## Data Availability

No datasets were generated or analysed during the current study.
